# Toward richer metadata for microbial sequences: replacing strain-level NCBI taxonomy taxids with BioProject, BioSample and Assembly records

**DOI:** 10.4056/sigs.4851102

**Published:** 2014-01-29

**Authors:** Scott Federhen, Karen Clark, Tanya Barrett, Helen Parkinson, James Ostell, Yuichi Kodama, Jun Mashima, Yasukazu Nakamura, Guy Cochrane, Ilene Karsch-Mizrachi

**Affiliations:** 1 National Center for Biotechnology Information, National Library of Medicine, National Institutes of Health, Bethesda, MD, USA; 2 European Molecular Biology Laboratory, European Bioinformatics Institute, Wellcome Trust Genome Campus, Hinxton, UK; 3 DDBJ Center, National Institute of Genetics, Research Organization for Information and Systems, Yata, Mishima, Japan

## Abstract

Microbial genome sequence submissions to the International Nucleotide Sequence Database Collaboration (INSDC) have been annotated with organism names that include the strain identifier. Each of these strain-level names has been assigned a unique ‘taxid’ in the NCBI Taxonomy Database. With the significant growth in genome sequencing, it is not possible to continue with the curation of strain-level taxids. In January 2014, NCBI will cease assigning strain-level taxids. Instead, submitters are encouraged provide strain information and rich metadata with their submission to the sequence database, BioProject and BioSample.

## Toward richer metadata for microbial sequences

The NCBI taxonomy database provides the organism nomenclature and classification that is used in sequence entries by the International Nucleotide Sequence Database Collaboration (INSDC [1]; comprising GenBank, ENA and the DDBJ) [2]. The NCBI Taxonomy Group is responsible for curating names for taxa that are regulated by the relevant codes of nomenclature [3-5], for providing informal names for specimens that are not identified with Linnaean species binomials, and for maintaining the ‘taxid’ namespace. This is a labor-intensive and largely manual effort undertaken by this small group of diligent and dedicated taxonomists at the NCBI [6].

It has been almost twenty years since the first bacterial genomes started to appear in the sequence databases, beginning with *Haemophilus influenzae* in 1995, followed within a year by *Escherichia coli*. In those days each new genome sequence was of significant scientific interest and represented a considerable technical achievement. At that time, for the convenience of those at INSDC institutes and their users, the taxonomy group started assigning strain-level taxids for prokaryotes with complete genome sequences, e.g.: “*Haemophilus influenzae* Rd” [7] and “*Escherichia coli* K12” [8]. (That genome is currently indexed as “*Escherichia coli* str. K-12 substr. MG1655”, since there are now many genomes sequenced from ‘strain’ K-12.) Since that time, the policy of assigning strain-level taxids for genome sequences has been extended to cover eukaryotic microbes as well – unicellular fungi, algae and protists – but it has never been applied to the multicellular eukaryotes. In particular, strain-level taxids have never been assigned for breeds of dogs, or for inbred strains of mice, or for individual human genomes.

Sequencing technology has undergone remarkable development over the past twenty years and it has become increasingly cheap and easy to sequence genomes, a trend that promises to continue in the foreseeable future. We are already seeing the submission of hundreds of genomes at a time that are simply time points of micro-evolutionary studies in *Escherichia coli*, or *Saccharomyces cerevisiae*. Another growing industry in genome submissions is in efforts to track epidemics, food-borne illnesses and hospital infection pathways. More will appear as this technology finds applications in other fields.

Our recognition that the curation of strain-level taxids will not remain possible under such growth, and that alternative data resources relating to biological samples are maturing at the INSDC partner institutes, has led us to a review of our practices in this area. We intend to discontinue the curation of strain-level taxids for microbial genomes submitted beyond January 2014. Importantly, this change in practice will not be applied retrospectively; we will not remove any of the thousands of strain-level nodes that we have added in the past, and we will continue to add informal strain-specific names for genomes from specimens that have not been identified to the species level, e.g.: “*Rhizobium sp.* CCGE 510” and “*Salpingoeca* sp. ATCC 50818”.

We strongly encourage submitters to annotate their genome submissions with the relevant source metadata, including strain, culture collection and isolation information as appropriate, plus the appropriate species (or subspecies) name. The Genomic Standards Consortium maintains checklists of Minimal Information about any (x) Sequence (MIxS) [9] that contain mandatory and optional descriptive metadata fields for a variety of organism types. These MIxS checklists can be included in the genome submission.

Our alternative system for recording and presenting strain-level annotation will be provided by the respective BioSample databases of the INSDC partner institutes [10-12]. BioSample records provide a single accessioned unit of information relating to a sample that has been assayed using sequencing or other platforms. This information serves to gather together taxonomic information, informal infraspecies information (such as strain), descriptors relating to the sampling process, accession information for the physical sample itself, etc.

For genome submissions, INSDC databases guide submitters through a series of logical steps in which the information required is requested and transferred. An early step is the registration of the initiative (BioProject) or indication that the genome data are connected to an existing initiative (This registration is applied within the INSDC host institutes’ respective BioProject and study databases). Following this, users are prompted to provide rich descriptive information about the sequenced sample(s) (BioSample) or an indication that samples already registered have been sequenced. Description of new samples, and updates and enhancements to existing samples, take advantage of defined checklists or ‘packages’ of attributes, appropriate for the initiative. In later steps of the genome submission process, users provide sequence data and functional annotation that connect to the samples described or selected.

BioSample records are one tool that can be used as an organizing and retrieval key to the genome datasets, as the strain-level taxid was in the past. BioSample accessions can be used to aggregate submitted data deposited in various archives, such as those that cover sequence (i.e. INSDC) and those that cover array-based studies (such as GEO, ArrayExpress and the DDBJ Omics Archive) [12-14]. The BioSample record will enable users to retrieve data across databases from samples with particular attributes. For instance, one may wish to retrieve submitted data for all *Salmonella enterica* strains isolated from a particular agricultural plant.

INSDC assembly records are another powerful tool in this area, as these hold the information about a particular genome assembly and are supported with unique assembly-level identifiers. In these records all of the pieces of a genome are collected together in ways that are much more flexible and powerful for indexing and retrieval purposes than were strain-level taxids. For example, genomes representing independent assemblies of the same sequence data share a BioSample accession, while those representing alternative sequencing studies of the same strain may have independent BioSample accessions.

The *Streptococcus pneumoniae TIGR4* (taxid 170187) genome initiative is described in PRJNA76613^2^. This record contains two genome assembles that were built from sequence reads from a single BioSample, SAMN00103527^3^. Two different assembly algorithms were used to create the assemblies, which are detailed in GCA_000269665^4^ and GCA_000273445^5^.

In an era when microbial genome sequencing was not as commonplace as it is now, using a taxid as a key to retrieve the genome and associated project metadata was a reasonable approach. However, with next-generation sequencing technology, one can sequence the genomes of hundreds of closely related microbes in a few hours [15]. Therefore, data consumers are better served by the new resources that we describe above that enable them to retrieve sets of genomes based on common attributes or initiatives.

The INSDC is prepared to stop assigning strain-level tax ids for strains of microbes that have their genome sequenced by January 2014 and encourages users to exploit other resources that allow them to explore sequence data by initiative, specimen or genome assembly.

## References

[1] INSDC http://www.insdc.org/

[2] Nakamura Y Cochrane G Karsch-Mizrachi I The International Nucleotide Sequence Database Collaboration. Nucleic Acids Res 2013; 41(D1):D21-D24 10.1093/nar/gks1084 23180798 PMC3531182

[3] Ride WDL, Cogger HG, Dupuis C, Kraus O, Minelli A, Thompson FC, Tubbs PK, eds. International Code of Zoological Nomenclature. 1999. 4th edn. International Trust for Zoological Nomenclature, The Natural History Museum, London http://www.nhm.ac.uk/hosted-sites/iczn/code/

[4] McNeill J Barrie FR Burdet HM Demoulin V Hawksworth DL Marhold K Nicolson DH Prado J Silva PC Skog JE International Code of Botanical Nomenclature (Vienna Code). Regnum Veg 2006; 146

[5] LaPage SP, Sneath PHA, Lessel EF, Skerman VBD, Seeliger HPR, Clark WA, eds. International Code of Nomenclature of Bacteria: Bacteriological Code (1990 Revision) 1992. ASM Press, Washington D.C. http://www.ncbi.nlm.nih.gov/books/NBK8817/21089234

[6] Federhen S The NCBI Taxonomy database. Nucleic Acids Res 2012; 40(D1):D136-D143 10.1093/nar/gkr1178 22139910 PMC3245000

[7] Fleischmann RD Adams MD White O Clayton RA Kirkness EF Kerlavage AR Bult CJ Tomb JF Dougherty BA Merrick JM Whole-genome random sequencing and assembly of *Haemophilus influenza* Rd. Science 1995; 269:496-512 10.1126/science.7542800 7542800

[8] Blattner FR Plunkett G III Bloch CA Perna NT Burland V Riley M Collado-Vides J Glasner JD Rode CK Mayhew GF The complete genome sequence of *Escherichia coli* K-12. Science 1997; 277:1453-1462 10.1126/science.277.5331.1453 9278503

[9] Yilmaz P Kottmann R Field D Knight R Cole JR Amaral-Zettler L Gilbert JA Karsch-Mizrachi I Johnston A Cochrane G Minimum information about a marker gene sequence (MIMARKS) and minimum information about any (x) sequence (MIxS) specifications. Nat Biotechnol 2011; 29:415-420 10.1038/nbt.1823 21552244 PMC3367316

[10] Barrett T Clark K Gevorgyan R Gorelenkov V Gribov E Karsch-Mizrachi I Kimelman M Pruitt K Resenchuk S Tatusova T BioProject and BioSample databases at NCBI: facilitating capture and organization of metadata. Nucleic Acids Res 2012; 40(D1):D57-D63 10.1093/nar/gkr1163 22139929 PMC3245069

[11] Gostev M Faulconbridge A Brandizi M Fernandez-Banet J Sarkans U Brazma A Parkinson H The BioSample Database (BioSD) at the European Bioinformatics Institute. Nucleic Acids Res 2012; 40(D1):D64-D70 10.1093/nar/gkr937 22096232 PMC3245134

[12] Kodama Y Mashima J Kaminuma E Gojobori T Ogasawara O Takagi T Okubo K Nakamura Y The DNA Data Bank of Japan launches a new resource, the DDBJ Omics Archive of functional genomics experiments. Nucleic Acids Res 2012; 40(D1):D38-D42 10.1093/nar/gkr994 22110025 PMC3244990

[13] Barrett T Wilhite SE Ledoux P Kim IF Tomashevsky M Marshall KA Phillippy KH Sherman PM Holko M Yefanov A NCBI GEO: archive for functional genomics data sets—update. Nucleic Acids Res 2013; 41(D1):D991-D995 10.1093/nar/gks1193 23193258 PMC3531084

[14] Rustici G Kolesnikov N Brandizi M Burdett T Dylag M Emam I Farne A Hastings E Ison J Keays M ArrayExpress update—trends in database growth and links to data analysis tools. Nucleic Acids Res 2013; 41:D987-D990 10.1093/nar/gks1174 23193272 PMC3531147

[15] Hu B Xie G Lo CC Starkenburg SR Chain PSG Pathogen comparative genomics in the next-generation sequencing era: genome alignments, pangenomics and metagenomics. Briefings in Functional Genomics. 2011; 10:322-333 10.1093/bfgp/elr042 22199376

